# Supplementary material for: Fault-tolerant quantum computation without distillation on a 2D device
Source: npj Quantum Inf. 2025 Nov 28;11(1):189. doi: 10.1038/s41534-025-01133-7 (PMC12662776; doi:10.1038/s41534-025-01133-7)
Supplement: Supplementary file 1 — Supplementary Information [file 41534_2025_1133_MOESM1_ESM.pdf]

Supplementary Material

## 1 Red Code Schedule

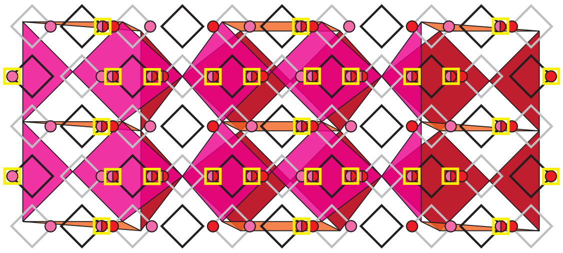

**Step 1:** Initialise all ancilla qubits in $|0\rangle$ (yellow boxes).

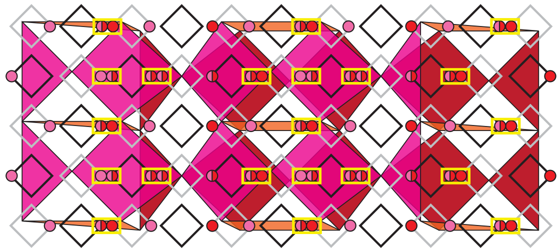

**Step 2:** All qubits move clockwise by one quater of a cycle. CNOTs are performed from data to ancilla qubits (yellow boxes).

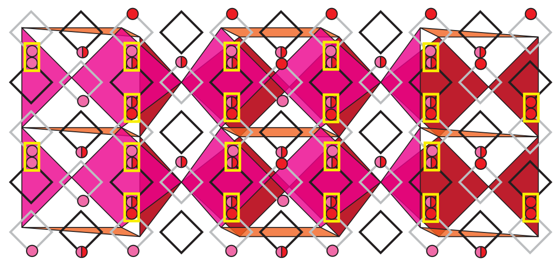

**Step 3:** All qubits move clockwise by one quater of a cycle. CNOTs are performed from data to ancilla qubits (yellow boxes).

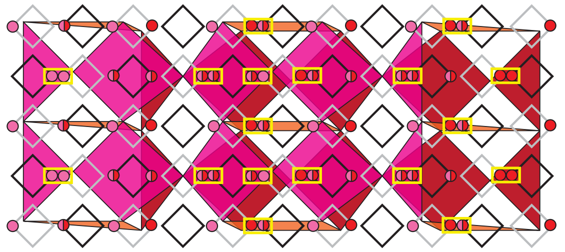

**Step 4:** All qubits move clockwise by one quater of a cycle. CNOTs are performed from data to ancilla qubits (yellow boxes).

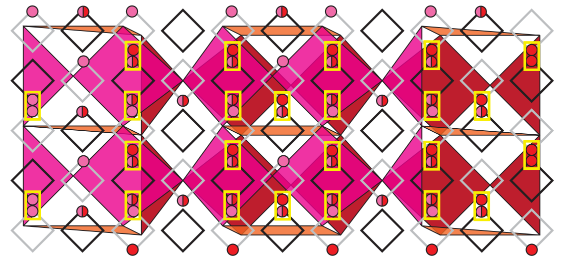

**Step 5:** All qubits move clockwise by one quater of a cycle. CNOTs are performed from data to ancilla qubits (yellow boxes).

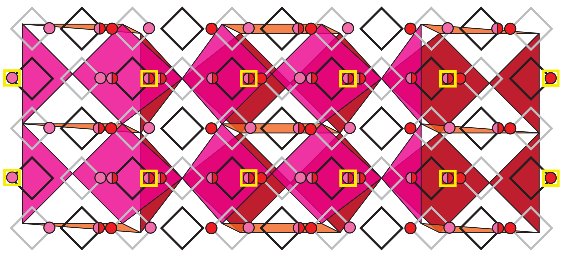

**Step 6:** Ancilla qubits which have completed all their interactions are measured out in $Z$ (yellow boxes).

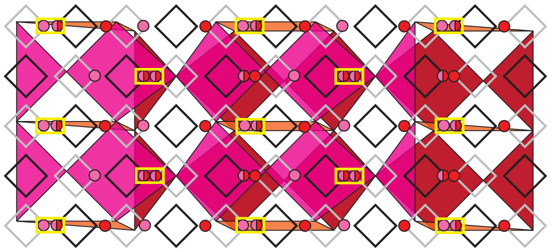

**Step 7:** Unmeasured ancilla qubits are advanced by half a cycle. CNOTs are performed from data to ancilla qubits (yellow boxes).

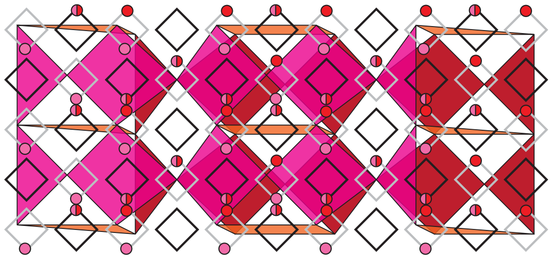

**Step 8:** All qubits move clockwise by one quater of a cycle. CNOTs are performed from data to ancilla qubits (yellow boxes).

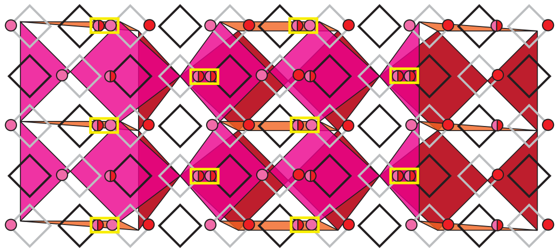

**Step 9:** All qubits move clockwise by one quater of a cycle. CNOTs are performed from data to ancilla qubits (yellow boxes).

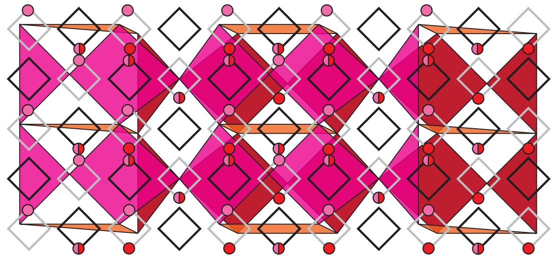

**Step 10:** All qubits move clockwise by one quater of a cycle. CNOTs are performed from data to ancilla qubits (yellow boxes).

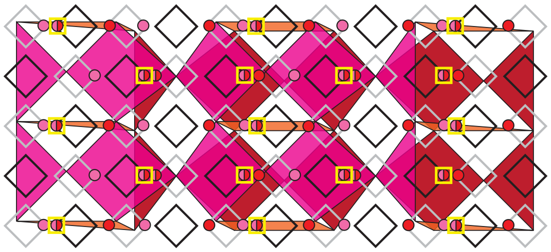

**Step 11:** Remaining ancilla qubits are measured out in $|Z\rangle$.

# 2 Blue Code Schedule

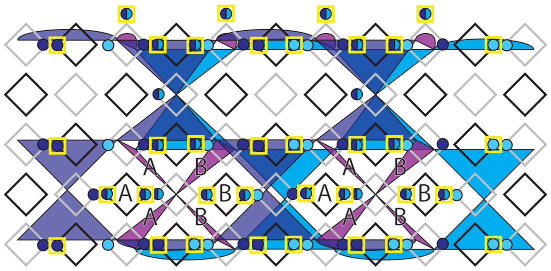


**Step 1:** Initialise all ancilla qubits in $|0\rangle$ (yellow boxes).

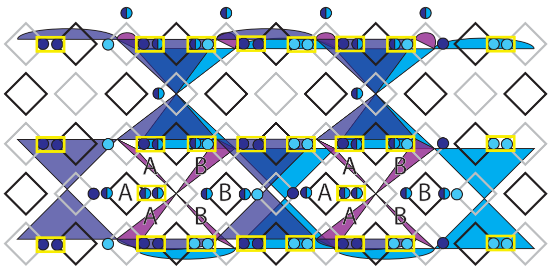


**Step 2:** All qubits move clockwise by one quater of a cycle. CNOTs are performed from data to ancilla qubits (yellow boxes).

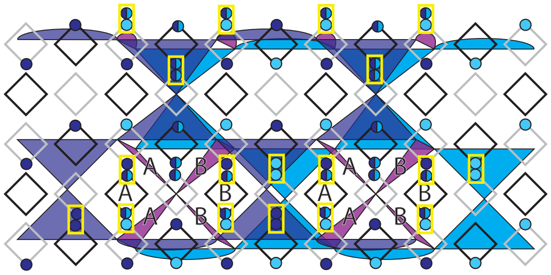

**Step 3:** All qubits move clockwise by one quater of a cycle. CNOTs are performed from data to ancilla qubits (yellow boxes).

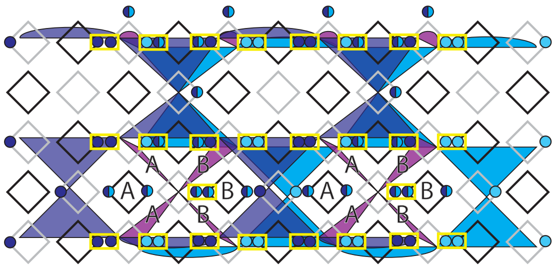


**Step 4:** All qubits move clockwise by one quater of a cycle. CNOTs are performed from data to ancilla qubits (yellow boxes).

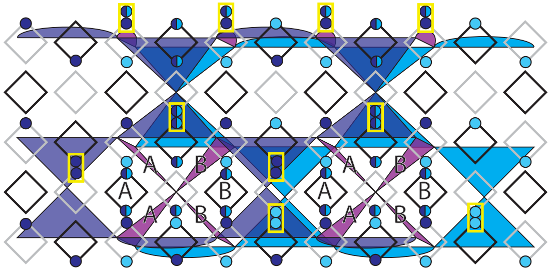


**Step 5:** All qubits move clockwise by one quater of a cycle. CNOTs are performed from data to ancilla qubits (yellow boxes).

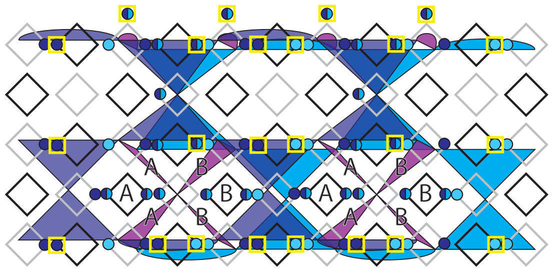


**Step 6:** Ancilla qubits which have completed all their interactions are measured out in $Z$ (yellow boxes).

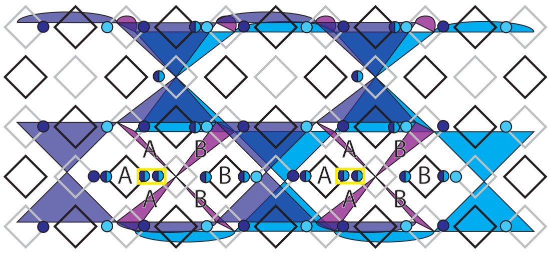


**Step 7:** Unmeasured ancilla qubits are advanced by half a cycle. CNOTs are performed from data to ancilla qubits (yellow boxes).

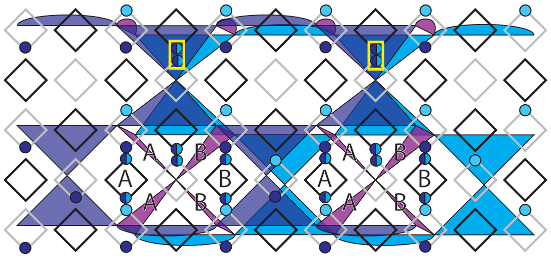


**Step 8:** All qubits move clockwise by one quater of a cycle. CNOTs are performed from data to ancilla qubits (yellow boxes).

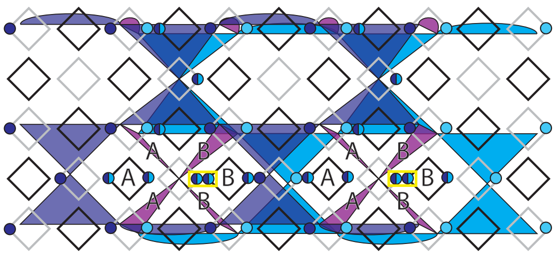


**Step 9:** All qubits move clockwise by one quater of a cycle. CNOTs are performed from data to ancilla qubits (yellow boxes).

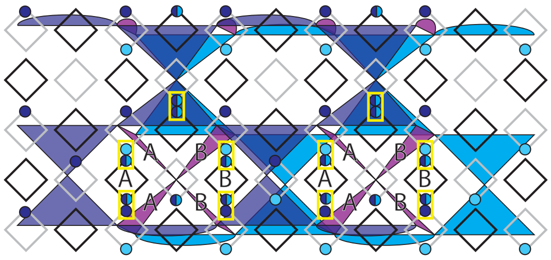


**Step 10:** All qubits move clockwise by one quater of

a cycle. CNOTs are performed from data to ancilla qubits (yellow boxes).

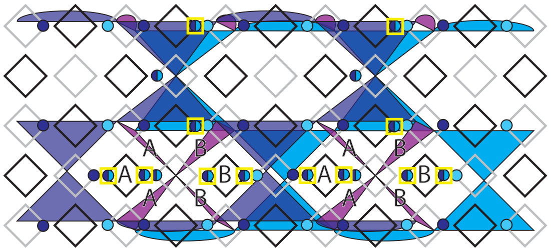

**Step 11:** Remaining ancilla qubits are measured out in $|Z\rangle$.

# 3 Green Code Schedule

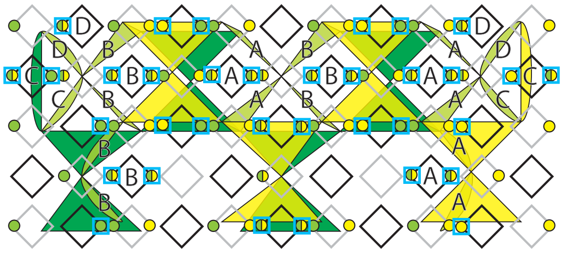

**Step 1:** Initialise all ancilla qubits in $|0\rangle$ (blue boxes).

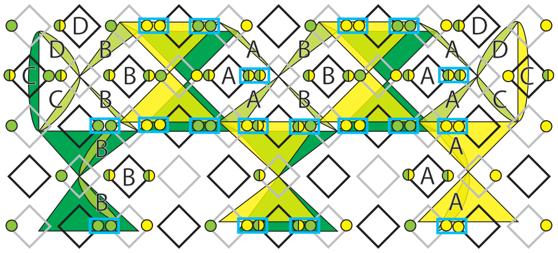

**Step 2:** All qubits move clockwise by one quater of a cycle. CNOTs are performed from data to ancilla qubits (blue boxes).

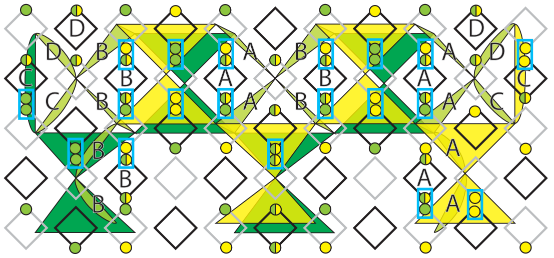

**Step 3:** All qubits move clockwise by one quater of a cycle. CNOTs are performed from data to ancilla qubits (blue boxes).

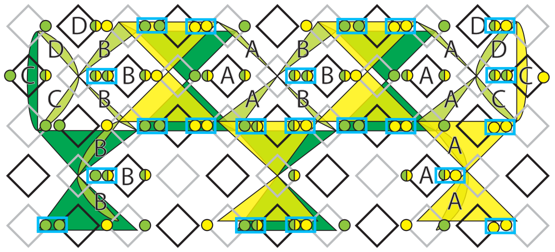

**Step 4:** All qubits move clockwise by one quater of a cycle. CNOTs are performed from data to ancilla qubits (blue boxes).

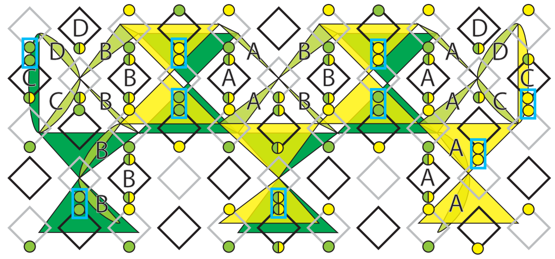

**Step 5:** All qubits move clockwise by one quater of a cycle. CNOTs are performed from data to ancilla qubits (blue boxes).

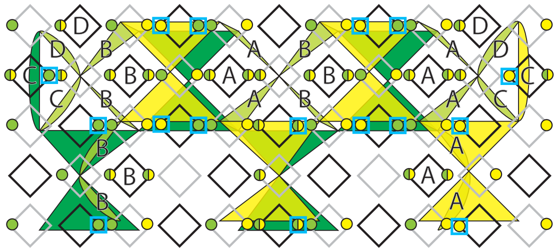

**Step 6:** Ancilla qubits which have completed all their interactions are measured out in $Z$ (blue boxes).

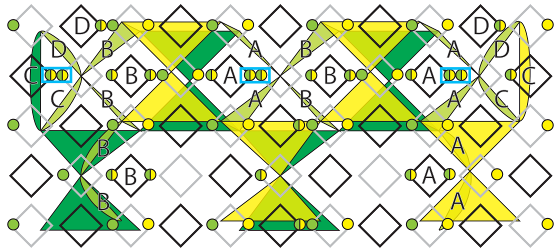

**Step 7:** Unmeasured ancilla qubits are advanced by half a cycle. CNOTs are performed from data to ancilla qubits (blue boxes).

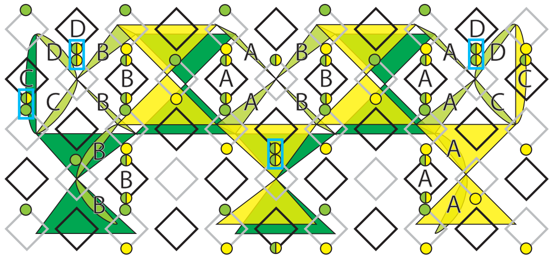

**Step 8:** All qubits move clockwise by one quater of a cycle. CNOTs are performed from data to ancilla qubits (blue boxes).

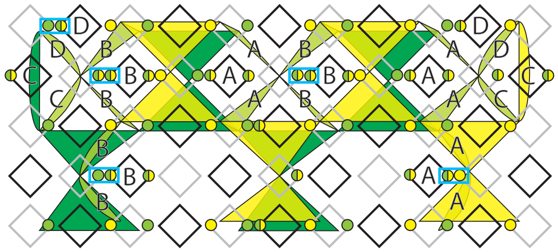

**Step 9:** All qubits move clockwise by one quater of a cycle. CNOTs are performed from data to ancilla qubits (blue boxes).

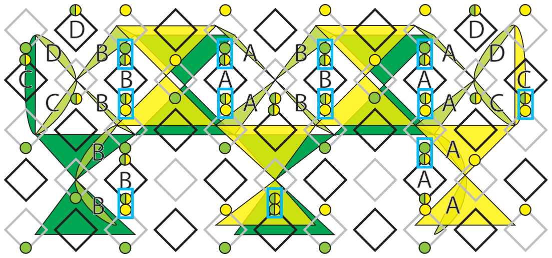

**Step 10:** All qubits move clockwise by one quater of a cycle. CNOTs are performed from data to ancilla qubits (blue boxes).

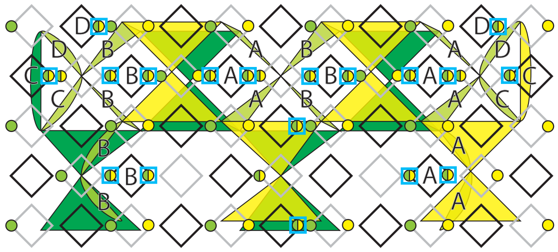

**Step 11:** Remaining ancilla qubits are measured out in $|Z\rangle$.
